# Observer Dreams: Criteria and Frequency

**DOI:** 10.1111/jsr.70201

**Published:** 2025-09-11

**Authors:** Darren M. Lipnicki

**Affiliations:** ^1^ Centre for Healthy Brain Ageing (CHeBA) UNSW Sydney Sydney Australia

**Keywords:** dream series, embodied, observer dream, participation, self

## Abstract

Numerous theories of dreaming consider embodied self‐representation and participation in dream events as key features. However, past studies suggest that the dream self is absent or an uninvolved observer in over 10% of adult REM dreams. Further, these dreams can be similarly elaborate and of comparable narrative structure to participatory dreams. The current study presents new criteria for observer dreams informed and illustrated by reports from adult dream series. Three types of observer status are described: (I) no embodied self‐representation, (II) an essentially disembodied self that sees events from within the dream environment, and (III) an embodied self that observes events virtually, often on TV. All types require observation of events or an activity with dynamic flow. The new criteria are used to determine observer dream frequencies in the dream reports from Hall and Van de Castle's 1963–1964 University of Miami study. The overall percentage of observer dreams in the Miami reports was 13.1%, and Type I was the most frequent (8.0% of all reports). Past studies show that observer dreams are more frequent in children and older adults than in young adults. An exploratory investigation of age differences in the current study was consistent with this, suggesting a rise in observer dreams from young adulthood. Additionally, direct physical aggression was found in around 20% of observer dreams, with potential implications for the threat simulation hypothesis of dream function. This study's findings suggest that observer dreams warrant greater consideration, including integration into theories of dreaming.

## 1 Introduction

Embodied self‐representation and participation in dream events are considered to be key features of dreaming in Domhoff's (2022) neurocognitive theory of dreaming, as well as the threat simulation hypothesis of dream function (Revonsuo 2000) and the NEXTUP model of dream function (Zadra and Stickgold 2021). While most rapid eye movement (REM) dreams do indeed feature a participatory dream self, sometimes the dream self is absent or an uninvolved observer of the events. Using samples of REM dreams from mainly young adults, Strauch and Meier (1996) found that there was no dream self in 10.6% of 198 dreams in one analysis, and 3.6% of 500 dreams in another. Snyder's (1970) study of 635 REM dreams from 58 young adults found that the self was absent in 5%, and that in 6% the self was present only as an observer, rather than a participant. Dreams with an uninvolved dream self who observes events from within the scene were reported to comprise an additional 7.6% of Strauch and Meier's sample of 198 REM dreams, and 6.7% of 90 REM dreams from 90 university students in a separate study by Occhionero et al. (2005). Looking across these studies suggests that around 11.7% of young adult REM dreams are observer dreams, with 5.3% having no dream self and 6.4% with a dream self who observes but does not participate in the dream events.

How observer dreams or dreamers not participating in events relate to theories of dreaming predicated on embodiment and participation has been addressed by the authors of these theories. In terms of his neurocognitive theory of dreaming, Domhoff (2022) says “the defining feature of dreaming is therefore the sense of being a participant in—*or more rarely, an observer of* [emphasis added]—an ongoing event.” (p. 53). The dreamer being an observer more rarely than a participant is consistent with Domhoff's summary statement that in dreams with social interactions “the dreamer is part of these social interactions about 80% of the time and an observer of them in the remaining 20%.” (p. 73). See Domhoff (1996) for further details on the normative percentages of witnessed vs. dreamer‐involved aggressions, friendliness, and sexuality from which this summary statement was derived. These normative data also show that 67.2% of dream reports feature at least one social interaction (Domhoff 2022), suggesting that the dreamer is merely an observer of social interactions in 13.4% of dreams. This is roughly in keeping with the reported percentages for observer dreams cited above, allowing for incomplete overlap with Domhoff's classification when observer dreams do not feature social interactions and dreams where social interactions are witnessed but the dreamer participates in other events.

Revonsuo (2005), in defining threatening dream events for the threat simulation hypothesis of dream function, specifies that “The definition does not give the dream self any special role in dream threats. Thus, movielike dream threats in which the self is not involved in any way are in principle possible” (p. 217). However, he further notes that “the dream self plays the starring role in threatening dream events” (p. 217), consistent with the notion that “the biological function of dreaming is the simulation of threatening events and the repeated rehearsal of threat perception and threat avoidance responses” (Revonsuo and Valli 2000, 3). Nevertheless, Revonsuo (2005) sees observer dreams as important for understanding aspects of selfhood and consciousness, as do other philosophers of mind, including Metzinger (2013) and Windt (2015). Finally, in explaining their NEXTUP model of dream function, Zadra and Stickgold (2021) posit that “Although NEXTUP arguably could create useful narrative simulations … without [self‐representation and embodied presence], such simulations would be like passively watching movies, and they would lack the verisimilitude created by this powerful combination” (p. 144).

The aim of the present study is to raise the profile of observer dreams, which seems warranted on two accounts: (1) the literature suggesting that at least 1 in 10 adult REM dreams is an observer dream, and (2) adult dream reports showing that observer dreams can be as elaborate and narratively well‐structured as any embodied and participatory dream. In attempting to fulfil this aim, observer dreams are explored here quantitatively after a qualitatively informed account of criteria development. The criteria were developed from readings of adult dream series and are illustrated by example reports from these series. The criteria are intended to be more definitive than previous categorisations, and to capture different types of observer status commonly seen in adult dream reports. The new criteria are applied in determining the percentage of observer dreams in a novel sample of dream reports, and for which dream reports from Hall and Van de Castle's 1963–1964 University of Miami study (Hall 1966) were used. Benefits of this sample include the well‐controlled collection of both laboratory‐based and non‐laboratory dreams from the same participants. This permitted a determination of observer dream frequency in non‐laboratory dream reports, which does not appear to have been done previously, and to compare this with the frequency of observer dreams in laboratory reports.

The Miami study data also permitted some exploratory investigations. The first of these involved age differences in observer dream frequency, given some evidence suggesting that older adults have a greater frequency of observer dreams than young adults. A study of dream reports from Navajo men aged 55–95 years classified the dreamer as passive in 35% and absent in 26% (Krohn and Gutmann 1971), consistent with another study of dream reports from men aged 60–71 years noting that “the most significant and consistent feature […] was the relatively passive participation of the dreamer” (Weisz 1969, 267). The second exploratory investigation stems from Revonsuo and Valli (2000) finding some dreams where the self could not react to a threat because they were not present in the scene. One of the more frequent and potentially severe threats in dreams is direct physical aggression, particularly in men's dreams (Revonsuo and Valli 2000). All Miami study participants were men and, considering potential implications for the threat simulation hypothesis of dream function, the frequency of direct physical aggression in their observer dream reports was determined.

## 2 Methods

### 2.1 Observer Dream Criteria

Observer dreams are defined here as dreams with events or an activity with dynamic flow in which the dreamer is not a participant, though they may have corresponding thoughts or emotions. Non‐participation is a corollary of the dreamer having either no self‐representation or an effectively disembodied point of view from within the dream environment, or of the events being observed virtually. These conditions categorise observer status as Types I, II, and III, respectively. An observer status need not be held for an entire dream for it to qualify as observer. The criteria addressing these conditions are detailed below and summarised in Table 1.

**TABLE 1:** Observer dream criteria summary.

A dynamic flow of observed events	The dream report should describe observation of events or an activity with dynamic flow explicitly indicated or inherent.
Observer status type	
Type I: Disembodied external observer	The dreamer has no embodied self‐representation and seems to observe events from outside the dream environment.
Type II: Passive internal observer	The dreamer sees events from within the dream environment but makes no bodily action nor is acted upon.
Type III: Embodied observer of virtual events	The dreamer is an embodied self‐observing virtual events, such as on TV.
Changes in observer status	An observer status need not be held for the entire dream. There may be changes to or from being an embodied participant.

Dreams used to illustrate aspects of the criteria, different observer status types, and the potential richness of observer dreams come from the author's dream series, as well as the publicly available and searchable dream series of others on DreamBank.net (Domhoff and Schneider 2008). The author's series contains thousands of dreams first recorded in early adulthood between 1990 and 1997, and then 2020 and present. Observer dreams were found on DreamBank by searching various series, particularly large series, using terms that included “observer” and “movie”. Where dream reports are cited, those from the author's series are labelled A1, A2, etc., and presented fully (with notes on type) in the Appendix, and those from DreamBank.net are labelled by the series name and number. The University of New South Wales Human Research Ethics Committee has acknowledged notification that this research uses data from a Publicly Available Dataset (Ref: iRECS6291).

#### 2.1.1 A Dynamic Flow of Observed Events

It is almost axiomatic in the neurocognitive theory of dreaming (Domhoff 2022), the threat simulation hypothesis (Revonsuo 2000) and the NEXTUP model (Zadra and Stickgold 2021) that the events in which an embodied dream self participates unfold in a narrative flow. This implies participation in a sequence of events or an activity with progression over time, a dynamic flow. Dynamic flow in a dream report may be explicitly indicated or inherent to an activity (e.g., sport is being played but play‐by‐play details are not indicated). As defined here, an observer dream (or dream portion) also has a sequence of events or an activity with progression over time, though in which the dream self does not participate. An observer dream report is thus distinguished from dream reports describing mere observation of a static image or scenario with no dynamic flow.

#### 2.1.2 Type I Observer Status

Type I is the quintessential observer status, with the dreamer having no embodied self‐representation and seeming to observe the events from outside the dream environment in which they occur. While this is sometimes explicitly indicated in a dream report (e.g., “I am watching as a disembodied observer”—Barb Sanders #2958), often there is simply no evidence of the dreamer being within the dream environment. Such dreams have been labelled “dreamer as observer” by DeGracia (1999) and described by Revonsuo (2005) as “… a simulation of the world unfolding in subjective consciousness. There are objects, persons, and events, but there is no representation of the dreamer present in that world simulation.” (p. 209). The contents of Type I dreams prove to be as varied as dreams with an active dream self, from the mundane (e.g., Appendix A1) to the adventurous and bizarre, as in this dream that includes thematically linked Type I scenes:

I see people outside, standing in an open area. Over a few times, they are pulled individually or in small groups by some force up into the sky, within a cloud formation. The speed at which they are pulled is immense, their trajectory like an exponential curve with a long tail just above the ground. The cloud is at least hundreds of metres away. […]

I now see a husband and wife who were part of the group, in their apartment. They are around 60 years old and wear very loose clothing, perhaps robes, and ornamental headwear that has a round part atop the head. These are the clothes they were returned in, not what they had on when taken. […]—Appendix A2.

For some further examples see Appendix A3 and Lavie and Wollman (1985), who describe an interesting case report of dreams with a Type I observer status that resemble very well formed and complete stories.

#### 2.1.3 Type II Observer Status

A dreamer with Type II observer status reportedly sees events from within the dream environment but is for all intents and purposes a disembodied self, making no bodily action nor being acted upon. Occhionero et al. (2005) describe this as “The dreamer is inside the scene, but totally a passive observer and no [sic] taking part in the oneiric scene.” (p. 80). This, and a previous similar description by Cicogna and Bosinelli (2001), implies that the dreamer is represented bodily in the dream environment. However, Windt (2015) suggests “that the feeling of self‐localization does not necessarily require the experience of oneself as a physical presence in the sense of being a phenomenally embodied self” and that without more details such instances represent “phenomenal indeterminacy with respect to the body” (p. 332). The reasonableness of this stance is demonstrated by dream reports of passive observation from within the dream environment that are ambiguous on embodiment, such as this example describing a point of view congruous with an embodied presence but with no direct evidence of embodiment: “I see all of this from behind and to the right of the people”—Appendix A4. In other reports, an embodied presence within the scene seems very likely excluded by the point of view described (e.g., “I see this as if from within a small computer monitor, an old type with depth, empty, through the glass.”—Appendix A5).

#### 2.1.4 Type III Observer Status

Type III observer status pertains to an embodied dream self‐observing virtual events, such as on TV or in an imaginary space. The dreamer does not participate in these events, and indeed has no practical means of doing so, but may interact with characters and other aspects of their local environment. The conditions of observation may be apparent from the start of a dream (e.g., “This is a television show that I see with my mom and sisters […]”—Kenneth #0634) or arise later (e.g., “[…] She causes an image to appear next to her about a metre high and nearly two metres across. She just seems to open up space into another dimension. The borders of this image shimmer blue and white. […]”—Appendix A6).

#### 2.1.5 Changes in the Dreamer's Status

An observer status is often not present or maintained for an entire dream but rather held by the dreamer for a portion or portions of a dream. Changes in either direction between observer and embodied participant in events are sometimes clearly indicated, as in these Type I examples: “I then entered the scene”—Van #088 or “I seem to be an observer rather than a participant now also”—Elizabeth #0522. When a change between observer status and embodied participant is not explicitly mentioned in a dream report, the presence of the observer portion may be easily overlooked. This is particularly likely when the sections are thematically linked or the latter section references something from an earlier section, as in both the Van #088 and Elizabeth #0522 dream reports cited above.

In some observer dreams the dreamer experiences multiple observer status types. It is also possible for a statement in a dream report to create ambiguity in how an earlier or current observer status type should be categorised, as in this example where a long seemingly Type I scene could be considered as having been Type III all along: “[…] This now seems like the end of a TV show episode. I turn to [Name], thinking that after that experience I don't know if we'll be watching the next one.”—Appendix A7.

### 2.2 Potential Additional Forms of Observer Dreams

Observer status Types I‐III do not address previously reported “observer” conditions where either a dreamer sees themselves within the dream environment from a third person perspective (Foulkes and Kerr 1994; Occhionero et al. 2005) or where dream events are seen vicariously, from the eyes of a character not identified as the self (Occhionero et al. 2005; Rosen and Sutton 2013).

It is unclear to what extent third person perspective dreams of oneself conflate observation and participation, as per Revonsuo's (2005) view that “the dreamer is represented, on the one hand, as a participant in the dream action and, on the other hand, as a more or less external observer.” (pp. 210–211). Nevertheless, this double representation of the dreamer as both observer and participant could arguably be considered a supplementary form of Type I observer status. Reports representing these sorts of dreams in the author's and DreamBank.net series investigated are rare compared to those with observer status Types I–III (e.g., Barb Sanders #2430).

The extent to which the dreamer is not a participant in vicarious dream events is also unclear. One possibility raised by Rosen and Sutton (2013) is that “… the occupant of the first‐person perspective in vicarious dreams really is, despite all appearances, the dreamer.” (p. 1048). This could account for nominally vicarious dreams in which the dreamer lacks clarity and reports being simultaneously both a non‐self‐character and themselves, but seeing from the character's eyes, as in these example dream portions:

[…] I am down here, or I see from a worker's eyes—the only one not blind. The others want to blind this person/me. A woman looks at them/me—her eyes have been altered by the powder and/or blindness—there seem to be more rings to her eyes, around the pupil, and a small white dot in the pupil. One of the rings or irises is light blue. She seems intent on getting the powder in “my” eyes and I look to run, though do not know if I can get away from the 4 or 5 workers here. […]—Appendix A8.

[…] The man is a fugitive! He runs through yards and streets. People chase him, and fall behind as he outruns them. He runs on a street. I see through his eyes. I may be he. […]—Kenneth #1418

Unlike in these examples, a dreamer could conceivably see vicariously through the eyes of a totally passive character, and their point of view would be compatible with a Type II observer status.

### 2.3 Determination of Observer Dream Percentages in Laboratory and Non‐Laboratory Dreams

The percentage of dreams featuring observer status Types I–III was determined for the sample of Hall and Van de Castle 1963–1964 University of Miami study (Hall 1966) dream reports available on DreamBank.net. The reports comprise both laboratory‐based REM and non‐laboratory dreams from 12 men aged 19 to 38 (median = 21) years old, with 11 providing 274 laboratory dreams (6–39 reports per participant) and 10 participants (9 the same) providing 172 non‐laboratory dreams (11–30 reports per participant). The laboratory dream reports were obtained from participants under four conditions: awakened once a night during an REM period, awakened during every REM period across the night, awakened during a time of adjustment to the laboratory, and when waking spontaneously during the night or remembering the dream in the morning. Not all conditions are represented in the reports of all participants. The dream reports were classified as observer (and type) or participant by the author first in January 2024, and then again 1 year later, in January 2025, without reference to the first classifications. Temporal reliability of the criteria could thus be assessed, as done similarly by Domhoff et al. (2006) for the coding of various dream content elements (not observer status). For the analyses, dream reports with classification disagreements were re‐evaluated and assigned a final classification.

### 2.4 Exploratory Investigations

The percentage of observer dreams in combined laboratory and non‐laboratory conditions was determined for two older (37–38 years old) and for nine younger (19–22 years old) Miami study participants. One participant aged 26 years was omitted to enable a clear distinction between the age groups. The frequency of direct physical aggression in observer dreams was also determined.

### 2.5 Statistical Analysis

The temporal reliability of the classifications made in 2024 and 2025 was assessed two ways: (1) with the proportion of agreement, calculated as the total number of agreements divided by the total number of reports classified, and (2) with the Intraclass Correlation Coefficient (ICC), using the two‐way mixed effects, absolute agreement, single rater/measurement approach recommended by Koo and Li (2016). Linear mixed models were used to determine whether the proportion of observer dreams (and their types) differed between laboratory and non‐laboratory dream reports. These models can accommodate some participants not providing reports for either the laboratory or non‐laboratory condition. Analyses were conducted using IBM SPSS Statistics 27.

## 3 Results

Dream reports that did not depict events with dynamic flow (either observed or participated in), had insufficient detail to determine observer or participant status, or seemed to describe one of the potential forms of observer dreams not included in the criteria (third person or vicarious) were excluded. There were 2 non‐laboratory and 17 laboratory dreams excluded, leaving 170 non‐laboratory dream reports and 257 laboratory dream reports available for analysis. In assessing the temporal reliability of the ratings, across the two rating periods there were 47 agreements on observer status, 368 agreements on participatory status, and 12 disagreements, giving the proportion of agreement = 0.97. The intra‐rater reliability analysis found ICC = 0.87 (95% confidence interval = 0.85–0.89), indicating good reliability (Koo and Li 2016) between classifications made 1 year apart.

All observer status Types I–III were found in the Miami study dream reports, and as found in reports from dream series, some of the Miami reports mentioned being a mere observer of events, with one even demarcated into “observation” and “participation” sections (Sam‐H‐7). Across all Miami study participants, there were 35 laboratory observer dreams (13.6%) and 18 non‐laboratory observer dreams (10.6%). The overall percentage of observer dreams was 13.1%, with the primary or most characteristic observer status type being Type I for 34 (8.0% of all dreams), Type II for 9 (2.1%), and Type III for 10 (2.3%). The numbers of each type are shown separately for the laboratory and non‐laboratory dreams in Table 2. All participants but one reported at least one observer dream, with the frequency range across all participants being 0%–25.0%.

**TABLE 2:** Numbers of observer status Type I, II, and III and participant dreams in the Miami study non‐laboratory and laboratory dream reports.

Dream report source	Dreamer status, *n* (%)			
	Observer			Participant
	Type I	Type II	Type III	
Non‐laboratory (*N* = 170)	12 (7.1)	4 (2.4)	2 (1.2)	152 (89.4)
Laboratory				
All (*N* = 257)	22 (8.6)	5 (2.4)	8 (3.1)	222 (86.4)
REM awakenings (*N* = 196)	19 (9.7)	3 (1.5)	4 (2.0)	170 (86.7)

The mixed model analyses showed that observer dreams were significantly less frequent than participant dreams (mean difference = −15.18, 95% CI = −18.63 to −11.74, *p* < 0.001), and that Type I observer dreams were reported more frequently than Type II (mean difference = 1.17, 95% CI = 0.29–2.06, *p* = 0.012) and Type III (mean difference = 1.14, 95% CI = 0.21–2.06, *p* = 0.018). There was no difference in the proportion of observer dreams between laboratory and non‐laboratory reports (status by condition interaction: estimate = 4.36, 95% CI = −2.52 to 11.25, *p* = 0.198). There were also no differences in the proportion between laboratory and non‐laboratory reports for any of the observer types (Type I: estimate = 4.18, 95% CI = −2.68 to 11.04, *p* = 0.213; Type II: estimate = 4.93, 95% CI = −1.83 to 11.69, *p* = 0.139; Type III: estimate = 4.45, 95% CI = −2.32 to 11.23, *p* = 0.180).

To facilitate comparison with past studies reporting percentages of observer dreams for laboratory‐based REM dreams, Table 2 shows the number of observer dreams for each type in the 196 Miami study dream reports on Dreambank.net obtained after REM awakenings (i.e., no adjustment or spontaneous dreams). These reports were from eight participants, and among them there were 26 observer dreams (13.3%).

The exploratory investigation of age differences in observer dream frequency involved determining the percentage of observer dreams for older (37–38 years old) and younger (19–22 years old) Miami study participant groups. As raw values, the frequency of observer dreams was higher for the older participants (15.8%; 15/95) than for the younger participants (11.1%; 33/298). Including a participant aged 26 years in the younger group made little difference to the result (11.4%; 38/332). Direct physical aggression was present in 18.9% (10/53) of the Miami study observer dream reports, or 20.8% (11/53) if including fatal aggression between animals. Direct physical aggression was present in all three observer dream types and involved a friend or relative of the dreamer in only two of the reports.

## 4 Discussion

The mean frequency of observer dreams found in the Miami study dream reports was as high as 13.6%, with Type I (disembodied external) observer status the most common type. The overall percentage of observer dreams in the REM dream reports is a little higher than the average of 11.7% for young adults calculated across previous studies (Occhionero et al. 2005; Snyder 1970; Strauch and Meier 1996). This could be because of a broader conception of observer dreams in the current study, which included Type III observer dreams (embodied observer of virtual events) and dreams with both observer and participant parts that previous studies did not explicitly address. The percentage of dreams featuring a Type I observer status sits in the overall range of 3.6%–10.6% of dreams with no dream self previously reported (Strauch and Meier 1996), but the percentage of dreams featuring a Type II (passive internal) observer status is lower than the average of 6.4% of dreams across three studies with a very similar categorisation (Occhionero et al. 2005; Snyder 1970; Strauch and Meier 1996). While Type III observer status was not explicitly addressed in previous studies, relevant dreams may have been captured by categories featuring a passive observer of events. Even so, adding the Type III dreams to the Type II total does not bring the Type II percentage to a level similar to that reported by previous studies, suggesting that other differences in the criteria or samples between studies are involved.

One possible difference in criteria or their use between the current and previous studies involves the classification of Type II observer status. Previous studies might have considered the following statement in one of the Miami reports as indicating self‐representation within the dream environment: “This dream started out, I was actually an onlooker, observer, through the whole thing.” (Erik‐S‐7). In the current study, such statements were considered ambiguous and potentially consistent with both Type I and Type II observer status. Without a definitive statement positioning the dream self, or at least the dreamer's point of view, within the dream environment during the observed events, these and other reports were deemed to be Type I. It should also be acknowledged that the current study's requirement for the observed events to depict or at least imply dynamic flow may have led to lower numbers of reports being classified as observer dreams relative to some previous studies, though noting that in Snyder (1970) the definition of a dream report included temporal progression or change in imagery.

In terms of samples, the previous studies reporting observer dream frequency featured men and women, while the Miami study featured only men. However, it is not known if observer dream frequency differs between men and women. The Miami study sample may also differ from previous study samples by including two participants older than young adults. Omitting these participants lowered the mean observer dream frequency to 11.4%, which more closely aligns with the 11.7% calculated across previous study results. However, Strauch and Meier (1996) describe the participants from which their sets of 198 and 500 dream reports were obtained as “mainly young adults” (p. xi), suggesting that some older adults were included.

Regardless of whether two middle‐aged participants were included or not, the prevalence of observer dreams for adults, found here and previously (Occhionero et al. 2005; Snyder 1970; Strauch and Meier 1996), is far less than that reported for children, and seemingly less than that reported for older adults. Foulkes et al. (1990) found that before eight years old at least half of children's dreams involve being a passive observer or have no self‐representation, but also that these dreams only featured static images or relatively simple narrative structures, unlike the elaborate nature of adult observer dreams demonstrated in the current study. Regarding older adults, one previous study reported that men aged 60–71 years were frequently passive in their dreams (Weisz 1969), while another found that Navajo men aged 55–95 years were passive or absent in 60% of their dreams (Krohn and Gutmann 1971). The current study's exploratory finding that middle‐aged adults had a slightly higher frequency of observer dreams than young adults is consistent with a rise in observer dream frequency from young adulthood to later life. It should be noted that Krohn and Gutmann's (1971) study also featured men aged 30–54 years, but the sample of dreams reported was too small to consider. Future studies with larger sample sizes and featuring both men and women from a broad age range are needed to reliably investigate changes in observer dream frequency in adulthood.

There was no significant difference in the frequency of observer dreams or their types found between laboratory and non‐laboratory dreams. Though based on a relatively small sample size, this finding is analogous to the general similarity of content found for laboratory and non‐laboratory dreams, as summarised by Domhoff (2022, 71–72), and both types of dream reports will be useful for future investigations of observer dreams. However, non‐laboratory reports from dream series may be the most useful for answering particular questions. The frequency of observer dreams among Miami study participants ranged from 0% to 25%, suggesting the possibility of individual differences in observer dream frequency beyond any effect of age. This would be in keeping with the large individual differences in dream content evident among dream series (see Domhoff 2022, chapter 4), which with their typically large number of reports per individual facilitate reliable results. Series have further shown how individual differences in dream content reflect continuity between dreaming and waking life (e.g., Bulkeley 2018; Markert and Schredl 2024). Whether this continuity is maintained in dreams where the dreamer does not participate in events or even feature is a fascinating question, yet just one of many questions about observer dreams that future analyses of dream series may seek to answer.

How do observer dreams fit within theories of dreaming that consider embodiment and participation as key features or otherwise integral to the process? Domhoff's (2022) neurocognitive theory of dreaming is independent of any possible adaptive function for dreaming, and within which the distinction between observer and participatory dreams is conceivably explained by different activation patterns in default mode network regions subserving self‐representation.

Revonsuo (2005) sees observer dreams as possible within the threat simulation hypothesis of dream function, consistent with finding threats in dreams where the dreamer was absent (Revonsuo and Valli 2000). The current study investigated direct physical aggression as a threatening event in observer dreams. At around 20%, the proportion of Miami study observer dreams with at least one direct physical aggression was higher than in Revonsuo and Valli's (2000) sample of all dreams, where direct physical aggression accounted for 17% of all threats among men but 33.6% of dreams did not have any threats. Further, it was mostly strangers involved in direct physical aggression in the Miami study observer dreams. Such dreams seem to be considered unable to serve any threat perception and avoidance rehearsal function (Revonsuo and Valli 2000). It can thus be asked if observer dreams with direct physical aggression (or other threats) can be accounted for in any way by the threat simulation hypothesis of dream function, or if they are seen as similarly extraneous as the reported third of all dreams with no threats.

Referring to their NEXTUP model of dream function, Zadra and Stickgold (2021) suggest that dreams without self‐representation and embodied presence would be like watching a movie and lack verisimilitude. Observer dream reports sometimes state something like “I can see it all as if it's a movie” (Alta #70), possibly as a post hoc means to explain the self's absence rather than truly describing the experience itself. These dreams are typically Type I observer dreams (with no self‐representation), which the criteria in the current study distinguish from Type III observer dreams, in which an embodied self‐representation is indeed watching events unfold often in a movie or on TV. The dreamer is an embodied self within the dream environment, but they make no responses that influence the flow of depicted events. There is contingently a similar absence of responses in observer dreams Type I and Type II (possible self‐representation but no embodied responses made). The NEXTUP model of dream function postulates that dreamer responses to a self‐generated internal world drive the narrative (Zadra and Stickgold 2021). In an observer dream, this self‐generated world is not influenced by the actions of an embodied dream self yet can exhibit a complex narrative that reports suggest are no different in verisimilitude to participatory dreams. While Zadra and Stickgold (2021) suggest that NEXTUP could create narrative simulations in the absence of an embodied self‐representation, the process is not elaborated.

In conclusion, the aim of this study was to raise the profile of observer dreams by developing comprehensive criteria for different types of observer dreams, and to use the criteria to determine the frequency of observer dreams in both non‐laboratory and laboratory‐based dream reports. With narratives as sophisticated as those of participatory dreams and a frequency of up to around 13%, observer dreams warrant consideration and accounting for by theories of dreaming, which will be made more comprehensive and explanatory as a result.

## Author Contributions

**Darren M. Lipnicki:** conceptualization, writing – original draft, formal analysis.

## Ethics Statement

The University of New South Wales Human Research Ethics Committee acknowledged notification that this research uses data from a Publicly Available Dataset (Ref: iRECS6291).

## Conflicts of Interest

The author declares no conflicts of interest.

## Supporting information


**Appendix A1–A8.** Supporting Information.

## Data Availability

The data used in this study are publicly available from DreamBank: https://dreambank.net/.
