# Supplementary material for: Observer Dreams: Criteria and Frequency
Source: J Sleep Res. 2025 Sep 11;35(3):e70201. doi: 10.1111/jsr.70201 (PMC13193353; doi:10.1111/jsr.70201)
Supplement: Supplementary file 1 — Appendix A1–A8. Supporting Information. [file JSR-35-e70201-s001.docx]

This appendix contains full reports of the eight dreams of the author cited in the article (A1-A8), with notes on observer dream type and relevant sections indicated.

**A1**

- Type I in its entirety.

I see a man with dark hair behind a table giving a craft lesson, as though for a recording. Before him is a sheet of cardboard, a lighter blue (sky blue). From a similar sheet he has cut a curvy, organic shape and glued this onto the bottom left corner (from my POV). Using offcuts from the sheet for the next step in what is the creation of paper. He has a large “sauce bottle” of white glue and applies a squiggle of this across the cardboard. He talks of holding the offcuts higher above this and snipping them very finally so they will fall like confetti onto the sheet, sticking to the glue, and of repeating this process a few times. The last step will involve something else to finish off the creation of a sheet of paper. I think about aspects of this I don't understand, including how the “confetti” will create layers when each time only a squiggle of glue is applied, and why in the final product there won't be a bulge where the cutout part was glued. As the man has been talking and demonstrating, his glue bottle and lots of other equipment has accumulated on the table, including on the sheet of blue cardboard. He has a small three -pronged tool, like a garden fork used with a raking action. It looks brand new, the prongs are dark blue metal with diamond-shaped heads. He leans forward with this, motioning to scrape back all of his equipment.

**A2**

- Type I in its entirety.

I see people outside, standing in an open area. Over a few times, they are pulled individually or in small groups by some force up into the sky, within a cloud formation. The speed at which they are pulled is immense, their trajectory like an exponential curve with a long tail just above the ground. The cloud is at least hundreds of metres away. I wonder if they are still alive, not believing the body capable of withstanding such acceleration and speed. The cloud now roils, a horizontal and vertical section, set against an otherwise clear blue sky. Soon something emerges, coming back the other way. A “plane” that is flat, like a cross section seen from above, though with a vertical tail. It is white or beige. All the people taken up into the cloud are sitting atop this, their arms up or out as if on an amusement park ride. I think the people must have experienced a different flow of time while “away”. I may think of TV shows where people go missing in weird circumstances and return en masse. The plane drops quickly to a city street, busy, quickly coming to rest in the middle of the road. I’m curious about how quickly it came to rest as this doesn’t seem possible given its speed in the air.

I now see a husband and wife who were part of the group, in their apartment. They are around 60 years old, and wear very loose clothing, perhaps robes, and ornamental headwear that has a round part atop the head. These are the clothes they were returned in, not what they had on when taken. They are hurrying about, one saying excitedly to the other that “I can’t believe this is happening”. Something is to happen soon, like a revelation to humanity, and they are hurrying to see this or play their role.

**A3**

- Type I in its entirety

A man, late middle-aged at least, with grey hair is in a room, in the middle of which is a brown, almost conical vault, head-high or so tall and not much more than a metre wide at the base. The sides are made of panels, maybe six of them complete this structure. One of these panels, facing a corridor into this room, has been broken off, by others. The man inspects this, and curious about the inside enters the vault, which seems empty. He looks at little round things, about 1 cm wide, at about eye level, around the inside perimeter. I realise before he does that these are cameras, and that he may be watched by the vault owners - mafia or similar. The man works for them, but should not be in here - for which he knows he will be punished, most likely killed, even though he was at first inspecting someone else’s attempt to break in. He prepares to face those that will be coming for him, hiding behind a central pillar in the vault, brandishing a gun with a very wide barrel, which he points towards the corridor, through the missing panel. The voices of two men approaching are heard, one asking the other if they think someone is inside. The man in the vault fires his gun with a bright flash - I think he should have waited as the men were not in sight yet and will now be sure someone is there.

**A4**

- Type II in its entirety, with the section indicating a particular viewpoint from within the dream environment in bold italics

Outside in a city region. A film set has been brought here, a platform about the size of a large room that has plants, park bench - it is a park scene but I wonder if there is enough vegetation to be convincing. It sits about 2 m high. Two people involved in making the movie sit side-by-side near the park scene. ***I see all this from behind and to the right of the people.*** Both are men. The one on the left hands the one on the right around four or five yellow masks that are meant to be moulds of actors or other famous peoples’ faces. The person is holding up one, inspecting it carefully - it is either meant to be a mould of Chris Rock's face, or he is to wear this mask in the movie. The masks don't really look like moulds of faces, they are about twice as long as a face is high, and are essentially rectangular hard plastic that has some slight twists and curvature. They don't look like faces, nor do they seem like they would fit on a face.

**A5**

- Type II in its entirety, with the section indicating a particular viewpoint from within the dream environment in bold italics.

An open plan office seemingly some years ago - with old furniture, 80s or so. Night. A young woman, Winona Ryder, and an older woman, middle-aged with blonde hair They are going to play an online game for the first time. The older woman is going to be the navigator of the streets they will explore. However, based on an interaction between the women before arriving at the office - also their first meeting - Winona doubts the woman's navigational abilities. The older woman assumes that she will quickly learn the street layout and will have no problems navigating. Winona sits in an armless office chair, she wears long shorts, khaki or olive. The other woman stands behind or beside her. ***I see this as if from within a small computer monitor, an old type with depth, empty, through the glass.*** The game is to start. The competitor is a young man, I know is now online and observing the women. He speaks to them, saying they will play three times: “One time for ….., one time for food, and one time for your lives” (the order of the first two and forgotten word may be reversed). When the man says the last part about playing for their lives his voice turns deeper and sinister. The women don't seem to fully understand, and don't realise that there is potential danger ahead.

**A6**

- Type III after participatory scenes, with the transition in bold italics.

I was in a shallow quarry which was a camping site, there were a few camps set up, and I had a backpack on. Suddenly three girls appeared and walked towards me, one had red hair and the one in the middle had dark hair and was wearing all white. All were attractive. For some reason I turned away and quickly followed a path out of the quarry walking under a few trees and stopping by a pool of water. The pool was part of a lake with a narrow opening to the lake nearly congested by reeds. Past the reeds on the lake I saw a powerboat turning with white foam coming from under it. The side of the pool was about a metre of red bank and built into this (sort of) was a kitchen. There was a table, with just the top above water pushed up against the bank and a few shelves built into the bank. There were kitchen utensils, wooden spoons, spatulas etc on the table and stuck in the bank and the whole “kitchen” had been burnt, everything was blackened by fire. I felt disgusted that someone could leave this like this, littering the bush.

Then the three girls walked down the path towards me in the same formation as before and the middle girl asked me “Why do you think I am essentially evil?”. I had no answer and then we were all standing in a modern kitchen with large black and large white floor tiles, checkered. The middle girl was standing by a fridge and the other two girls were now flanking me. The middle girl is trying to persuade us not to want to experience the evil world, saying that we do not understand what it means. The two girls seem to express some wish to do so but I have no such desire and start to feel a little worried and scared. The middle girl looks very troubled and suddenly screams “No”. Her voice becomes very deep and she sound as though she has been possessed by something. ***She causes an image to appear next to her about a metre high and nearly two metres across. She just seems to open up space into another dimension. The borders of this image shimmer blue and white.*** The image is that of a dormitory, polished wooden floor and a row of neat beds on the right. Beyond the beds is a doorway. Through this doorway comes something evil, a dark hunched shape looking almost insectile but the size of a human with large clawed feet. It moves very fast and is dragging a woman in a dress with it. The woman is struggling frantically but the creature is strong. It moves to the foot of one of the beds and drags a boy up towards it, to the end of the bed. The woman is this boy’s mother and then standing behind her the creature digs its clawed hands into her from behind and kills her. Then they all disappear, leaving the dormitory still for a while. Then the creature reappears in the doorway and does the same thing dragging a male this time. It pulls the boy out from the bed and puts his face near that of the male he brought in. The face of the male becomes that of the boy and the boy screams. The creature then puts a claw through the back of the male’s head, two claws poking out the eyes from within sending rich red blood flowing outwards. Then his face is ripped apart from within. The creature then puts its head partway through the hole it created in the male’s head and seems to be smiling.

**A7**

- This dream appears to be entirely Type I, though the concluding sentence in bold italics creates ambiguity and the potential for it to be classified as Type III.

A dirt street, medieval-looking, behind which are crowded, over-towering buildings, blue and indistinct. The lighting at the street is not bright. In the street are three men in period attire, artists, who have been tasked by Inquisition-looking figures in chairs to inspect three items. The artists are lying on the ground. The item one has is a piece of paper around half A4 size on which is a religious passage or psalm, in a number of small stanzas. However, most of these have been crossed out and alternative wording written nearby, maybe something to do with Islam. The man is copying everything to another piece of paper. The other two artists have different sorts of items. Going against the “Inquisition” all are fascinated by their items and wish to know more. They all roll on the ground, now covered in red paint, slyly looking at one another and putting a red finger mark of paint on a cheek – a secret signal of their desire to continue working with these items and to know more. This odd behaviour does not go unnoticed by the inquisitors however, and next I see one of the artists strung up, upside down, his head being dunked in a wooden basin on the ground. I don’t think the liquid is water, but something caustic or harmful. He is then bound to a chair and beaten in his face until unrecognisable, though wrapped in some white bandages at the end. His fellow artists may have been forced to do the beating. The other two are then treated similarly. Then a variety of cartoonish puppets go through the beating on the chair, often with something like a heavy and strong-bristled brush. For these characters it seems to be a birthday tradition, with the one whose birthday it is a willing participant – to be beaten. I see a close-up of a puppet’s face, purple, with a part like a sausage shape – he says “Happy birthday”. Then nearby, closer to a building is something like an ATM on which are numerous small puppets – cute looking. One is different to the rest. All face this way. ***This now seems like the end of a TV show episode. I turn to [name], thinking that after that experience I don’t know if we’ll be watching the next one.***

**A8**

- This example is not assigned a Type I, II or II classification. It may be an additional form of observer dream where the dreamer sees vicariously from a character’s eyes, with lines indicating this in bold italics.

A hill, at the base of which trucks deposit a white powder like talc or magnesium that has a slight blue tinge and some small balls a few mm in diameter that haven’t broken up. There is bushland about, a road that runs past the base of the hill. It is night. Migrant workers push the powder up the hill using their bare hands, to the top where it is processed. This leaves a wide trail of the powder up the hill. ***I see from a worker’s eyes, their hands shovelling the powder upwards.*** I am sure this must be toxic over time, exposure to bare skin, and believe the company knows this but doesn’t care. At the base of the hill a worker is saying they are blind, the powder having affected their eyes. It is a woman, who proceeds to throw powder into the faces of others, to blind them. I find her ability to do this conflicts with her claim to be blind. It is day. Other workers are now also blind, and act similarly to the first woman. ***I am down here, or I see from a worker’s eyes – the only one not blind.*** The others want to blind this person/me. A woman looks at them/me – her eyes have been altered by the powder and/or blindness – there seem to be more rings to her eyes, around the pupil, and a small white dot in the pupil. One of the rings or iris is light blue. She seems intent on getting the powder in “my” eyes and I look to run, though don’t know if I can get away from the 4 or 5 workers here. The area resembles the topography of [name’s] front yard – the hill and street also slightly uphill.
